# MicroRNA-365 promotes lung carcinogenesis by downregulating the USP33/SLIT2/ROBO1 signalling pathway

**DOI:** 10.1186/s12935-018-0563-6

**Published:** 2018-05-01

**Authors:** Yuhuan Wang, Shuhua Zhang, Hejing Bao, Shukun Mu, Baishen Zhang, Hao Ma, Shudong Ma

**Affiliations:** 1 Department of Oncology, Nanfang Hospital, Southern Medical University, Guangzhou, 510515 Guangdong China; 2 Department of Oncology, Chongqing Three Gorges Center Hospital, Chongqing, China; 3 Department of Clinical Medicine, Tianjin Medical University College, Tianjin, China

**Keywords:** Adenocarcinoma of lung, Carcinogenesis, Deubiquitinating enzymes, MicroRNAs, Oncogenes

## Abstract

**Background:**

Abnormal microRNA expression is closely related to cancer occurrence and development. miR-365a-3p plays an oncogenic role in skin cancer, but its role in lung cancer remains unclear. In this study, we aimed to investigate its role and underlying molecular mechanisms in lung cancer.

**Methods:**

Western blot and real-time quantitative PCR (qPCR) were used to detect the expression of miR-365a-3p in lung adenocarcinoma and lung cancer cell lines. The effects of miR-365a-3p on lung cancer cell proliferation, migration, and invasion were also explored in vitro. The potential miR-365a-3p that targets *USP33* was determined by dual luciferase reporter assay and verified by qPCR and western blot analysis. miR-365a-3p acts as an oncogene by promoting lung carcinogenesis via the downregulation of the miR-365a/USP33/SLIT2/ROBO1 axis based on western blot analysis. Subcutaneous tumourigenesis further demonstrated that miR-365a-3p promotes tumour formation in vivo.

**Results:**

miR-365a-3p was upregulated in lung adenocarcinoma and lung cancer cell lines. Overexpression of miR-365a-3p promoted and inhibition of miR-365a-3p suppressed the proliferation, migration, and invasion of lung cancer cells. We identified *USP33* as the downstream target of miR-365a-3p and observed a negative correlation between miR-365a-3p and *USP33* expression in lung adenocarcinoma patients. The miR-365/USP33/SLIT2/ROBO1 axis, a new mechanism, was reported to inhibit the invasion and metastasis of lung cancer. A nude mouse model of lung cancer further verified these findings.

**Conclusions:**

In summary, miR-365a-3p acts as an oncogene by promoting lung carcinogenesis via the downregulation of the USP33/SLIT2/ROBO1 signalling pathway, making the miR-365/USP33/SLIT2/ROBO1 axis a new mechanism of lung cancer promotion and a novel therapeutic target for predicting prognosis and response to gene therapy.

**Electronic supplementary material:**

The online version of this article (10.1186/s12935-018-0563-6) contains supplementary material, which is available to authorized users.

## Background

According to the World Health Organization (WHO), cancer is the leading cause of death worldwide, and lung cancer is the main cause of cancer-related deaths [1]. In 2017, there were an estimated 222,500 new diagnoses of lung cancer in the United States, and 155,870 people died of lung cancer [2]. With advances in medical technology, lung cancer treatment has entered the era of precision medicine [3–5]. Therefore, identifying genes that drive cancer progression and providing effective treatments may significantly prolong the survival of patients with non-small cell lung cancer (NSCLC) [6, 7].

MicroRNAs (miRNAs) are small, highly conserved, noncoding RNAs of approximately 19–25 nucleotides that regulate gene expression at the post-transcriptional level by base-pairing with the 3′ UTRs of target mRNAs [8–11]. They play key roles in human biological processes, such as migration, cellular metabolism, cell proliferation, apoptosis, and epithelial–mesenchymal transition (EMT) [12–17]. Increasing evidence has demonstrated that miRNAs play an important role in cancer and are closely related to tumourigenesis and prognosis. The miRNA miR-365 gene is located on chromosome 16p13.12, and the mature hsa-miR-365 sequence is cleaved from two precursors: hsa-miR-365-1 and hsa-miR-365-2 [18]. Abnormal expression of miR-365 is observed in a variety of tumours, with different expression patterns and functions in different human cancer types. In skin squamous cell carcinoma, we first found that miR-365a-3p plays an oncogenic role by downregulating NFIB to promote CDK6 and CDK4 expression, leading to Rb phosphorylation and tumour progression [18–20]. Additionally, high expression levels of miR-365a-3p can be detected in breast and pancreatic cancer, playing a role in promoting tumour development [21, 22]. In colon cancer, miR-365a-3p inhibits the development of cancer by targeting cyclin D1 and BCL-2 [23, 24]. This miRNA also serves as a tumour suppressor gene in gastric cancer [25]. In brief, the functions of miR-365a-3p in cancer are complex. Thus, the roles of miR-365a-3p in lung cancer cells remain unclear and require further study.

Deubiquitinating enzymes are key enzymes that can reverse ubiquitination modifications. Recently, their functions and mechanisms have been of great interest in tumour research [26, 27]. *USP33*, also known as VHL-interacting deubiquitinating enzyme 1 (*VDU1*), is located on chromosome 1 and encodes two protein subtypes: type I and type II. Type I contains 942 amino acids, and type II contains 911 amino acids, with predicted molecular weights of 107 and 103 kDa, respectively. USP33 has been confirmed to inhibit the development of a variety of tumour cells. USP33 deubiquitinates and thus maintains the stability of ROBO1, thereby regulating the activity of SLIT2 and inhibiting cancer cell metastasis [28–30]. USP33 maintains the stability of CP110 and antagonises the effect of cyclin-F on the S/G2 and G2/M phases of the cell cycle by interacting with CP110, maintaining the steady state of the centriole and ensuring normal functioning of mitosis and genome stability, which reduces tumourigenesis [31].

Additionally, SLIT2/ROBO1 signalling has been shown to inhibit tumour cell proliferation and migration. Prasad et al. [32] found that ROBO1 and ROBO2 were expressed in several breast cancer cell lines and that SLIT2 inhibited CXCL12/CXCR4-induced chemotaxis, invasion, adhesion, and secretion of MMP-2 and MMP-9 in breast cancer cells. The loss of SLIT2, SLIT3, or ROBO1 protein in mouse breast cancer models results in an increase in repair processes in tissues, promoting cell proliferation [33].

Our previous studies showed that in cutaneous squamous cell carcinoma, miR-365a-3p plays an oncogenic role by promoting tumour progression [18]. Moreover, studies have shown that USP33-mediated SLIT2/ROBO1 signalling participates in the development of cancer [29, 30]. Thus, in this study, we aimed to elucidate the role of miR-365a-3p in lung cancer cells and the relationship among miR-365a-3p, USP33, SLIT2, and ROBO1 in lung adenocarcinoma.

## Methods

### Tissue samples

This study was approved by the Ethics Review Board of Nanfang Hospital, Southern Medical University (Guangzhou, China). Twenty pairs of primary lung cancer tissues and their corresponding adjacent non-tumour tissue samples were collected in the Thoracic Surgery Department of Nanfang Hospital from March to June 2017. All experiments were performed according to relevant guidelines; informed consent was obtained from each patient. No patients received prior radiotherapy or chemotherapy. All specimens were removed during surgery and immediately stored at − 80 °C in liquid nitrogen for subsequent extraction of total RNA.

### Cell lines and cell culture

A549 cells were purchased from the American Type Culture Collection (ATCC, Manassas, VA, USA). SPC-A-1 and H1299 cells were purchased from the Shanghai Cell Bank of the Chinese Academy of Sciences (Shanghai, China). All cells were cultured in RPMI-1640 medium or DMEM supplemented with 10% FBS (Gibco, Gaithersburg, MD, USA). All cells were maintained in a humidified incubator at 37 °C and 5% CO_2_.

### RNA extraction and real-time quantitative RT-PCR (qRT-PCR)

Total RNA was extracted from cell lines or tissues using a TRIzol Kit (Takara Bio, Shiga, Japan) according to the manufacturer’s instructions. cDNA was synthesized using Takara RT reagent (Takara Bio). qRT-PCR was performed on a Light Cycler 480 system (Roche Diagnostics, Basel, Switzerland) using a SYBR Green I Master kit (Roche). We used glyceraldehyde-3-phosphate dehydrogenase (*GAPDH*) as an internal reference. Mature miR-365a-3p expression was measured by qRT-PCR according to the Taqman MicroRNA Assay protocol (Takara Bio) and normalised using *U6* small nuclear RNA with the 2^−ΔΔCt^ method.

### Western blotting

Equal amounts of protein were separated by 10% SDS-PAGE and blotted onto PVDF membranes (Millipore, Bedford, MA, USA) probed with the following primary and secondary antibodies: monoclonal rabbit primary antibodies against SLIT2 and ROBLO1 (1:1000; Cell Signaling Technology, Boston, MA, USA) and β-tubulin (1:10,000; Bioworld Technology Inc., St. Louis Park, MN, USA); polyclonal rabbit primary antibody against USP33 (1:500; Abcam, San Francisco, CA, USA); and secondary fluorescent goat anti-rabbit antibody (LI-COR, Lincoln, NE, USA). Primary antibodies were applied overnight at 4 °C, and secondary antibody treatment was performed for about 1 h at 25 °C. An Odyssey Infrared Imaging System (LI-COR) was used to analyse immunoreactive bands. Western blotting was performed three times.

### Plate clone formation assay

A549 or SPC-A-1 cells were seeded into a 6-well culture plate (200 cells/well) and incubated for 12 days. Cells were stained with Giemsa solution. Plates were scored by determining the number of colonies containing ≥ 50 cells.

### 5-Ethynyl-2ʹ-deoxyuridine (EdU) assay

5-Ethynyl-2ʹ-deoxyuridine incorporation assays were conducted using the EdU assay kit (RiboBio Co., Guangzhou, China) according to the manufacturer’s instructions. Cells were incubated with 50 nM EdU for 2 h at 37 °C. Cells were then fixed with 4% formaldehyde for 15 min at 25 °C and treated with 0.5% Triton X-100 for 20 min at 25 °C to permeate cell membranes. After washing with PBS three times, cells were incubated with 1× Apollo reaction cocktail (100 µL/well) for 30 min. DNA was stained with 10 µg/mL of Hoechst 33342 stain (100 µL/well) for 20 min, and staining was visualised with fluorescence microscopy. Five fields of view were randomly selected for each sample. EdU-positive cells were stained with red dye, and the relative proliferation-positive ratios were calculated from the average cell count of the five visualised fields.

### Cell migration and invasion assays

The migratory and invasive abilities of cells were assessed using Transwell inserts (Corning, Inc., Corning, NY, USA) in 24-well plates. For invasion assays, each group of cells (5 × 10^4^ cells/100 µL) was resuspended in FBS-free RPMI-1640 medium and seeded into the upper chamber containing a Matrigel-coated membrane. After incubation for 24 h at 37 °C with 5% CO_2_, the incubation medium and non-invading cells were removed from the upper surface of the membrane with cotton swabs. Invading cells that adhered to the lower surface of the chamber were fixed in 4% paraformaldehyde for 20 min and stained with 0.1% crystal violet for 30 min. Invading cells were photographed and manually counted at 200× magnification using a microscope (Olympus, Tokyo, Japan). For the Transwell migration assays, the process was the same, except the Transwell membrane was not precoated with Matrigel. Each assay was performed at least three times independently.

### Wound healing assay

When cells had grown to approximately 90% confluency (after 48 h), an artificial wound was created with a 10-µL pipette tip. Cells were then cultured in fresh medium without FBS. Images were taken at 0 and 36 h to visualise wound healing. The relative percentage of the wound healed was calculated using the following formula: (width of wound at 0 h − width of wound at 36 h)/width of wound at 0 h.

### Plasmid and oligonucleotide construction

AntagomiR-365, antagomiR-negative control (antagomiR-NC), agomiR-365, and agomiR-negative control (agomiR-NC) were designed and synthesized by GenePharma (Shanghai, China). pcDNA3.1-USP33 and pcDNA3.1 vectors were designed and synthesized by Obio Technology (Shanghai, China).

### Transient transfection

A549 and SPC-A-1 cells were seeded in 6-well plates at a density of 30–50%. Transient transfection was performed with Lipofectamine 2000 reagents (Invitrogen, Carlsbad, CA, USA) according to the manufacturer’s instructions. For all experiments, cells were collected 24–28 h after transfection.

### Dual-luciferase reporter assay

The dual-luciferase reporter plasmids psi-CHECK2-USP33 (containing the wild-type *USP33* 3ʹ UTR binding site) and psiCHECK2-mGPC3 (containing a mutant *USP33* 3ʹ UTR) were constructed. A549 or SPC-A-1 cells were added to 24-well plates at 70–80% confluence 24 h before transfection. A mixture of 50 nM miR-365a-3p agomiR or antagomiR and 0.5 µg psi-CHECK2 reporter plasmid (psiCHECK2-wUSP33 or psiCHECK2-mUSP33) was co-transfected into cells using Lipofectamine 2000 reagent. At 48 h after transfection, luciferase activity was analysed using a dual-luciferase reporter assay system (Promega, Madison, WI, USA) according to the manufacturer’s instructions. Each experiment was performed in triplicate.

### In vivo tumourigenesis assays

All animal experimental protocols were approved by the Animal Research Ethics Committee of Nanfang Hospital and complied with the rules of the specific pathogen-free (SPF) animal laboratory of the Nanfang Medical University. 4- to 6-week-old male mice were purchased from the Animal Laboratory Center of Nanfang Medical University (Guangdong, China). A549/antagomiR-365 cells and A549/antagomiR-NC cells were injected subcutaneously into the left and right axilla of five nude mice (4 × 10^6^ cells on each side) beginning 8 days after the injection of tumour cells. The mice were sacrificed 26 days post-injection to observe changes in tumour volume. Tumour size was measured every 3 days using the same protocol, and tumour volumes were calculated with the following formula: *V* = (*L* × *W*^2^)/2, where *V* is the volume (mm^3^), *L* is the biggest diameter (mm), and *W* is the smallest diameter (mm).

### Bioinformatics analysis

The Cancer Genome Atlas (TCGA) data portal provides a platform for researchers to query, download, and analyse data sets generated by TCGA (http://cancergenome.nih.gov/).

Bioinformatics analysis performed with miRanda/TargetScan/starBase indicated that the 3′ UTR of *USP33* is a binding site for miR-365a-3p.

### Statistical analysis

Data are expressed as mean ± SD. Each experiment was repeated at least three times unless otherwise indicated. Statistical analyses were performed using SPSS 22.0 software (SPSS, Chicago, IL, USA). Significant differences were analysed using Student’s *t* tests for continuous variables. Spearman’s correlation was used to analyse the relationship between miR-365a-3p and *USP33* mRNA expression; *P* < 0.05 indicated significance.

## Results

### miR-365a-3p is upregulated in NSCLC tissues and cell lines

**Fig. 1:**
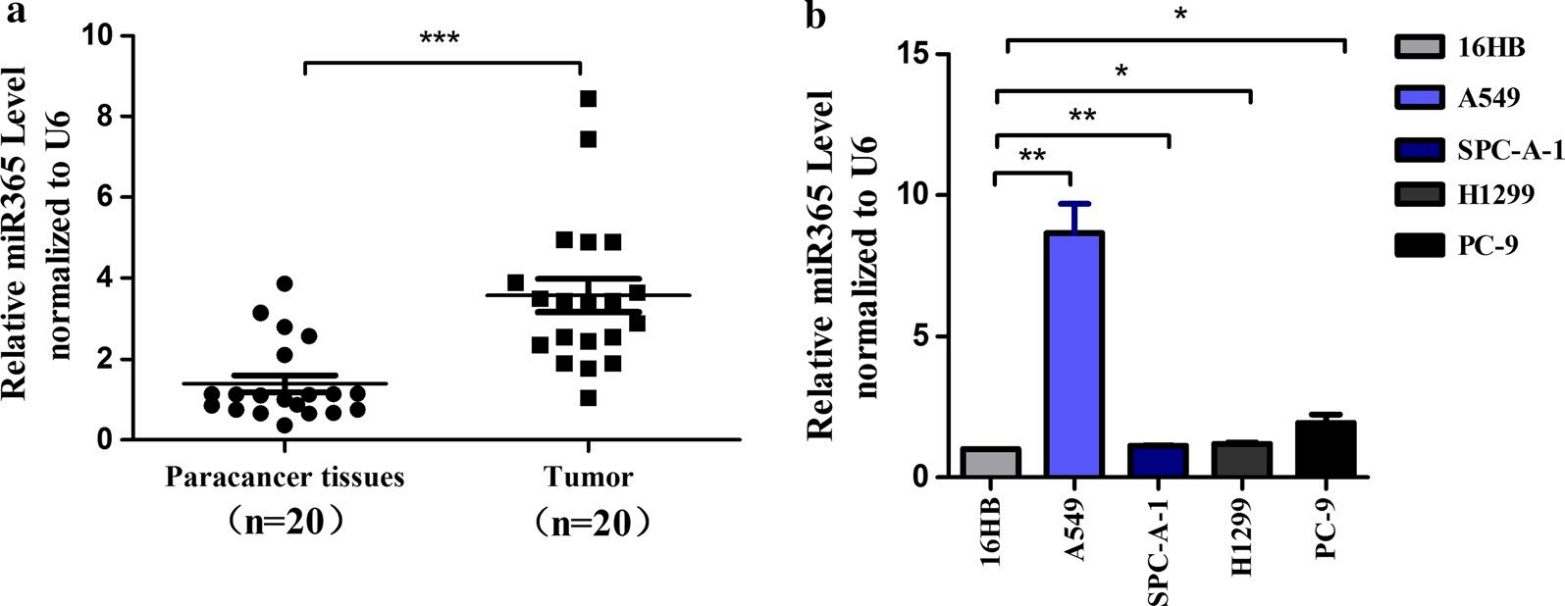
miR-365a-3p is upregulated in non-small cell lung cancer tissues and cell lines. **a** Relative expression levels of miR-365a-3p were analysed by qRT-PCR in 20 paired human lung adenocarcinoma tissues and adjacent matched normal paracancerous tissues (n = 20 per group). **b** Relative expression levels of miR-365a-3p were analysed by qRT-PCR in the normal lung epithelial cell line 16HBE and lung cancer cell lines A549, SPC-A-1, and H1299. Results were normalised with *U6* in each sample. Experiments were repeated at least three times independently. Data represent mean ± SD. ***P < 0.001

To evaluate the expression of miR-365a-3p in lung adenocarcinoma cells, qRT-PCR assays were performed in 20 patients with lung adenocarcinoma and matched paracancerous tissues. *U6* was used as an internal control. Results showed that the average expression level of miR-365a-3p in cancer tissues was higher than that in the corresponding paracancerous tissues (fold difference = 2.560, *P* = 0.0329; Fig. 1a). Next, the expression levels of miR-365a-3p were analysed in various lung cancer cell lines. qRT-PCR results demonstrated that higher expression levels of miR-365a-3p were present in A549, SPC-A-1, H1299, and PC9 lung cancer cell lines than in 16HBE normal lung epithelial cells. This was consistent with our clinical findings, indicating increased miR-365a-3p expression in lung adenocarcinoma tissues (Fig. 1b). Additionally, among the lung cancer cell lines, miR-365a-3p exhibited the highest expression in A549 cells and the lowest expression in SPC-A-1 cells; therefore, we selected these two cell lines for subsequent experiments. Taken together, the above results indicated that the abnormal expression of miR-365a-3p may be involved in lung carcinogenesis.

### miR-365a-3p promotes proliferation, migration, and invasion in lung cancer cells

**Fig. 2:**
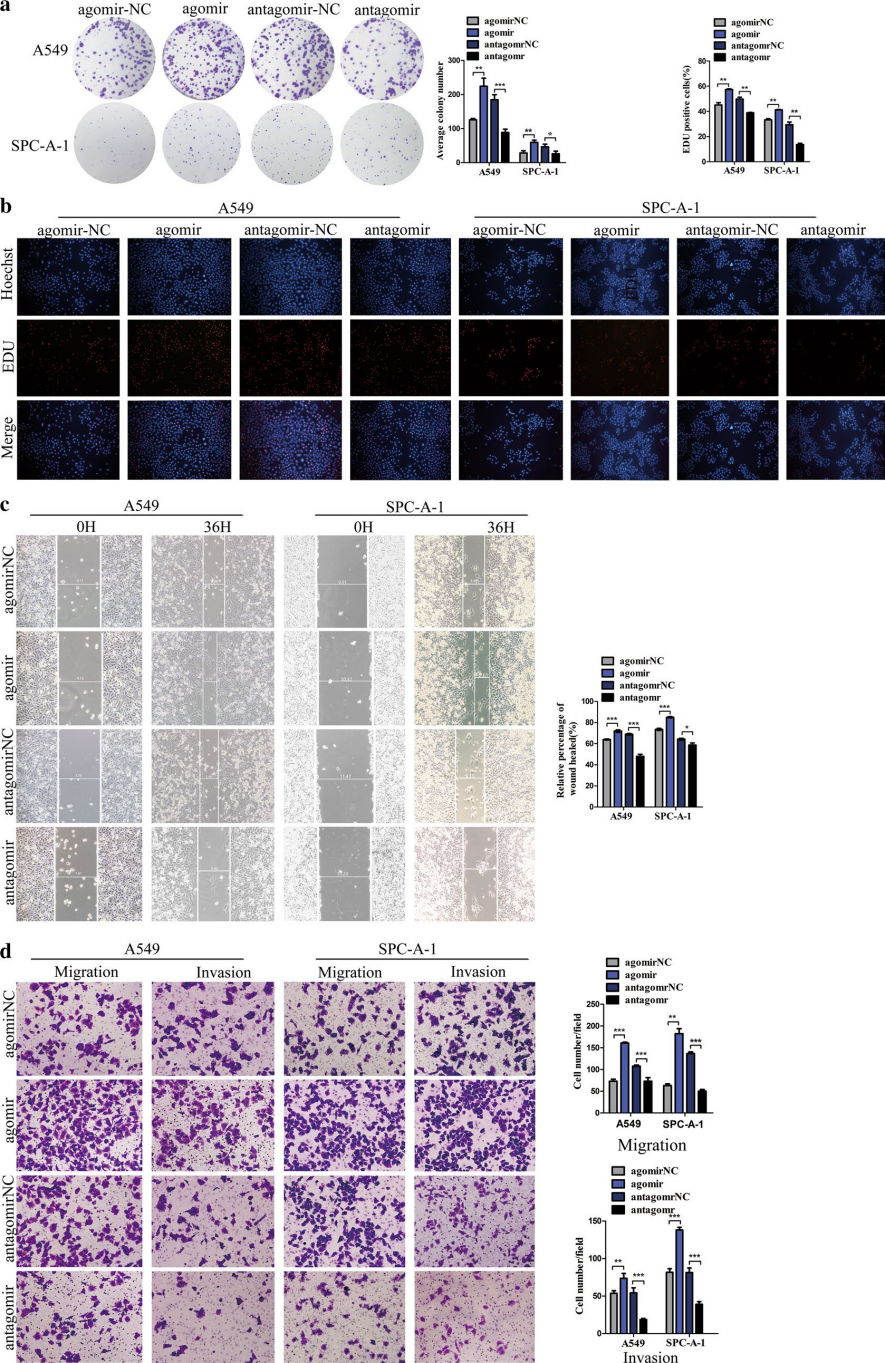
miR-365a-3p promotes the proliferation, migration, and invasion ability of lung cancer cells. **a** Plate clone formation efficiencies of A549 or SPC-A-1 cells following overexpression of miR-365a-3p by agomiR or inhibition of miR-365a-3p by antagomiR treatment, as compared with controls. **b** Representative images of A549 or SPC-A-1 cells stained with Hoechst (blue) and EdU (red), which indicates DNA synthesis, after transfection with agomiR-365, antagomiR-365, or controls. **c** Wound healing assay in A549 or SPC-A-1 cells transfected with agomiR-365, antagomiR-365, or controls. **d** Transwell migration and invasion assay in A549 or SPC-A-1 cells transfected with agomiR-365, antagomiR-365, or controls. For all experiments, agomiR-NC and antagomiR-NC were used as the negative controls for agomiR-365 and antagomir-365, respectively. Left panel, representative images. Right panel, quantitative data. All experiments were performed in triplicate and repeated three times independently. *P < 0.05, **P < 0.01, ***P < 0.001 compared to controls

Our findings suggested that the dysregulated expression of miR-365a-3p may result in lung cancer progression. To explore the function of miR-365a-3p, A549 and SPC-A-1 cells were transfected with agomiR-365 or antagomiR-365 (Additional file 1). To investigate the effect of high expression of miR-365a-3p on the proliferation of lung cancer cells, we used plate clone formation and EdU assays. The results of the plate clone formation assay showed that the overexpression of miR-365a-3p increased the number of colonies (*P* < 0.05), whereas the opposite result was observed following the inhibition of miR-365a-3p expression (*P* < 0.05; Fig. 2a). The EdU assay results revealed that cellular fluorescence intensity was increased after transfection with agomiR-365 (*P* < 0.05) and decreased after transfection with antagomiR-365 (*P* < 0.05; Fig. 2b). Thus, the EdU assay results were consistent with those of the plate clone formation assay, further confirming that miR-365a-3p promotes the proliferation of lung cancer cells.

Next, we performed wound healing and Transwell migration and invasion assays. The wound healing assay demonstrated that wound healing rates were elevated following transfection with agomiR-365 (*P* < 0.05) and reduced following transfection with antagomiR-365 (*P* < 0.05; Fig. 2c). In the Transwell migration and invasion assay, the numbers of cells that migrated and invaded the Matrigel were elevated following transfection with agomiR-365 (*P* < 0.05) and reduced following transfection with antagomiR-365 (*P* < 0.05; Fig. 2d). Thus, the results of the Transwell migration and invasion assay were consistent with those of the wound healing assay, indicating that miR-365a-3p promotes the migration and invasion activities of lung cancer cells.

### *USP33* is a downstream target gene of miR-365a-3p and is downregulated by miR-365a-3p

**Fig. 3:**
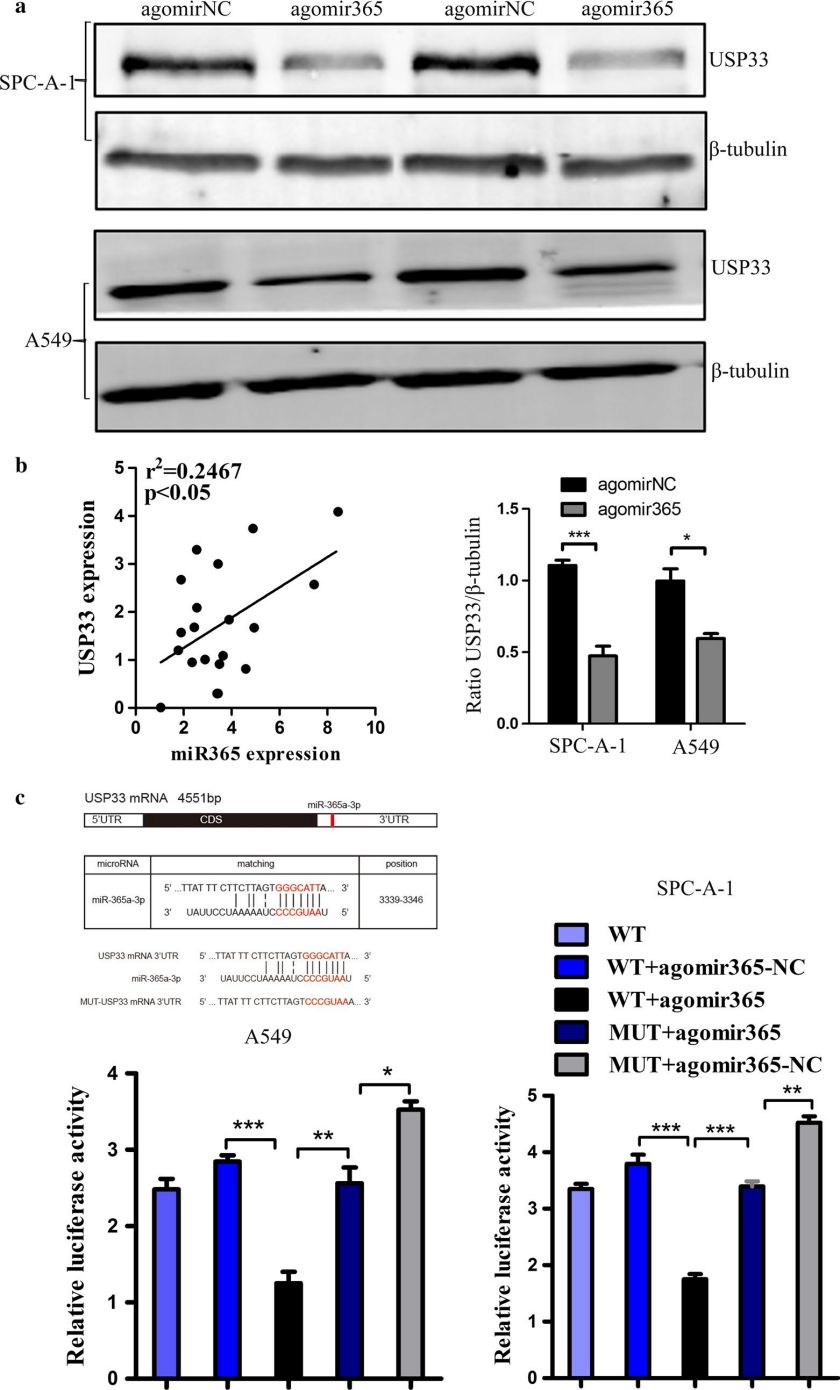
*USP33* is a downstream target gene of miR-365a-3p and is downregulated by miR-365. **a** Expression levels of USP33 were detected by western blotting of A549 or SPC-A-1 cells after transfection with agomiR-365, antagomiR-365, or controls. β-tubulin was used as an internal control. Experiments were performed at least three times, and representative bands are shown. **b** (a) Sequence alignments of miR-365a-3p binding sites in the wild-type and mutant *USP33* 3ʹ UTRs. The replaced site is underlined. (b) Wild-type *USP33* 3ʹ UTR or mutant *USP33* 3ʹ UTR was co-transfected with agomiR-365 or agomiR-NC into A549 or SPC-A-1 cells. After 48 h, relative luciferase activities were measured. *P < 0.05, **P < 0.01, ***P < 0.001. **c** Expression levels of miR-365a-3p and *USP33* were examined by qRT-PCR in 20 paired human lung adenocarcinoma tissues and adjacent normal lung tissues. A correlation was observed between miR-365a-3p and *USP33* expression as determined by Spearman correlation analysis (n = 20; *R*^2^ = 0.2467, *P* < 0.05)

The above in vitro experiments demonstrated that miR-365a-3p is closely correlated with the proliferation, migration, and invasion of lung cancer cells. Analysis using multiple databases showed that *USP33* may be a target gene of miR-365a-3p. TCGA data showed a negative correlation between miR-365a-3p and *USP33* expression, and survival analysis demonstrated that high expression of *USP33* was associated with good prognosis (Additional file 2). Additionally, it was previously reported that *USP33* exhibits low expression in lung adenocarcinoma tissues and cells [30, 34]. Thus, we sought to determine whether miR-365a-3p interacts with USP33. A western blot assay showed that the expression of USP33 was significantly downregulated following the overexpression of miR-365a-3p (Fig. 3a). Next, qRT-PCR was used to detect the expression of miR-365a-3p and *USP33* in tissues derived from 20 cases of lung adenocarcinoma. Consistent with our hypothesis, we observed a negative correlation between the expression of miR-365a-3p and that of *USP33* in these tissues (*R*^2^ = 0.2467, *P* < 0.05; Fig. 3b). To further verify that miR-365a-3p directly targets and downregulates *USP33*, the wild-type 3′ UTR of *USP33* (WT *USP33* 3′ UTR) or a mutated *USP33* 3′ UTR (Mut *USP33* 3′ UTR) was cloned into a dual-luciferase UTR vector and then co-transfected with agomiR-365 or a negative control (agomiR-NC) into A549 and SPC-A-1 cells. At 48 h after transfection, cells were harvested and lysed to detect luciferase activity (Additional file 3, Additional file 4: Table S1). The results showed that relative luciferase activity was significantly decreased in cells co-transfected with WT *USP33* 3′ UTR and agomiR-365. However, there was no significant change in luciferase activity after co-transfection with Mut *USP33* 3′ UTR and agomiR-365 (Fig. 3c). This suggested that miR-365a-3p directly targets and binds the *USP33* 3′ UTR.

### miR-365a-3p promotes lung cancer cell proliferation, migration, and invasion by downregulating *USP33*

**Fig. 4:**
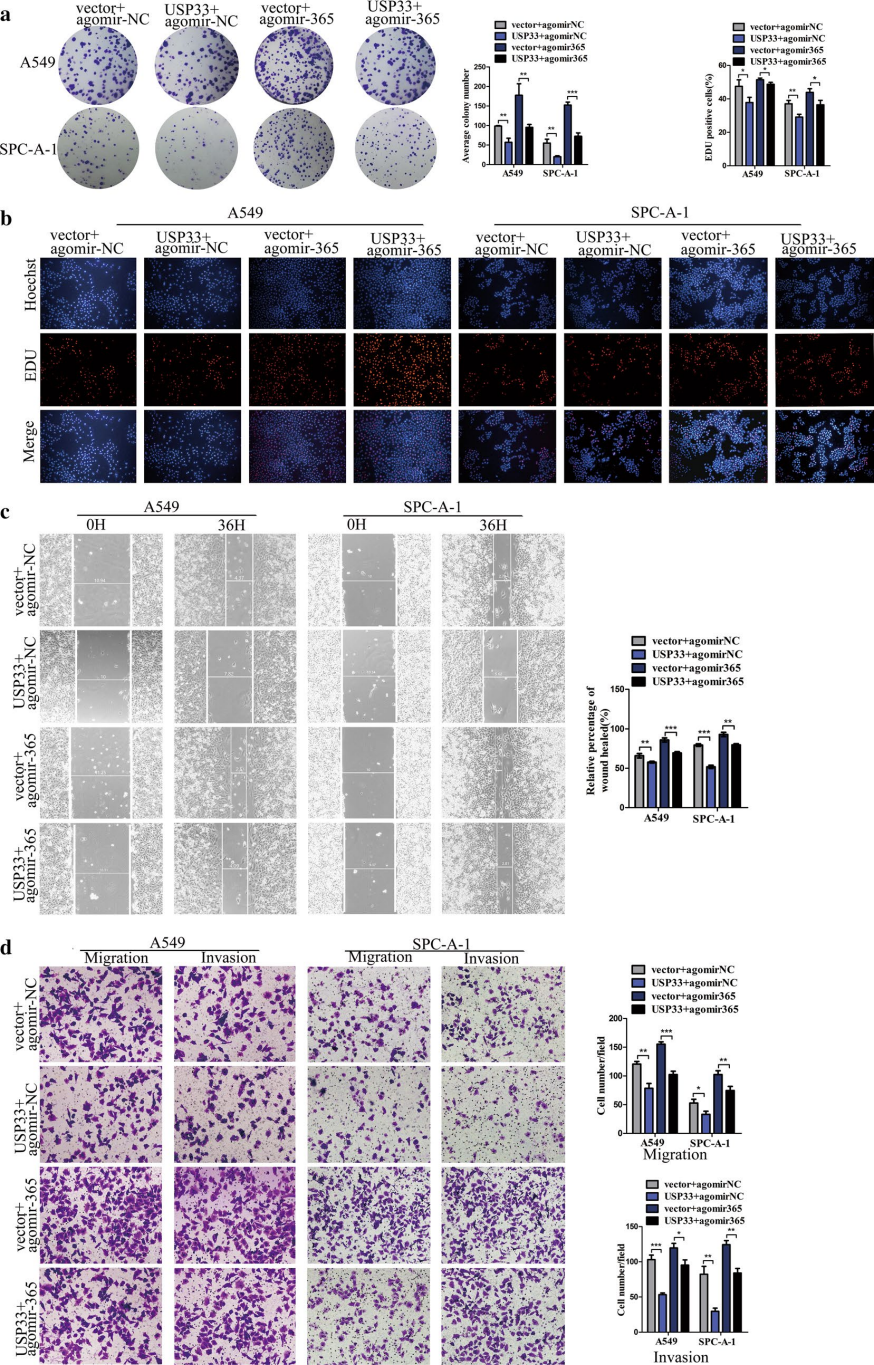
miR-365a-3p promotes lung cancer cell proliferation, migration, and invasion by downregulating *USP33*. **a**, **b** The proliferative ability of A549 or SPC-A-1 cells after overexpression of *USP33* by transfection of pcDNA3.1-USP33, miR-365a-3p by transfection with agomiR, or *USP33* and miR-365a-3p simultaneously was measured using the **a** plate clone formation and **b** EdU assays. pcDNA3.1-vector and agomiR-NC were used as negative controls. **c**, **d** The migration and invasion activities of A549 or SPC-A-1 cells transfected with pcDNA3.1-USP33, agomiR-365, or both vs. controls were measured using the **c** Transwell migration and invasion and **d** wound healing assays. Left panel, representative images. Right panel, quantitative data. All experiments were performed at least in triplicate and repeated three times. *P < 0.05, **P < 0.01, ***P < 0.001 compared to controls

The above experiments demonstrated that miR-365a-3p targets *USP33* and downregulates its expression. Next, functional rescue assays were performed to further verify that the miR-365a-3p-mediated downregulation of *USP33* promotes the proliferation, migration, and invasion of lung cancer cells. The pcDNA3.1-USP33 plasmid was constructed to overexpress *USP33*, with the empty pcDNA3.1 vector used as a negative control (Additional file 1b). The plate clone formation assay results showed that the numbers of cell colonies derived from A549 and SPC-A-1 cells co-transfected with pcDNA3.1-USP33 and agomiR-NC were significantly reduced (*P* < 0.05), whereas these numbers increased markedly compared with those in negative controls after co-transfection with pcDNA3.1 vector and agomiR-365 (*P* < 0.05). In contrast, the simultaneous overexpression of *USP33* and miR-365a-3p by co-transfection with pcDNA3.1-USP33 and agomiR-365 resulted in no significant changes in the number of cell colonies compared to that in the control group (Fig. 4a). The EdU assay results revealed that the overexpression of USP33 in combination with the overexpression of miR-365a-3p mitigated an increase in the cellular fluorescence intensity caused by the overexpression of miR-365a-3p alone, consistent with the results of the plate clone formation assay (Fig. 4b). This further suggested that miR-365a-3p promotes the proliferation of lung cancer cells by downregulating *USP33*. Similarly, the wound healing assay showed that the wound healing rate increased in cells overexpressing miR-365a-3p alone and decreased in the presence of pcDNA3.1-USP33 (Fig. 4c). The same patterns were observed in the Transwell migration and invasion assay (Fig. 4d).

### miR-365a-3p regulates the USP33/SLIT2/ROBO1 signalling pathway

**Fig. 5:**
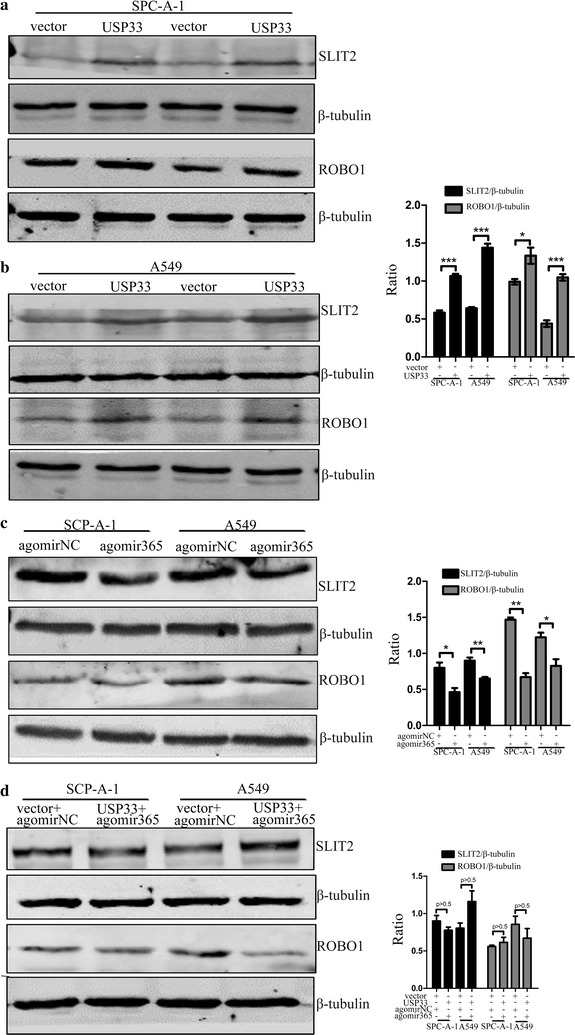
miR-365a-3p regulates the USP33/SLIT2/ROBO1 signalling pathway. Western blot was used to measured SLIT2 and ROBO1 expression levels following overexpression of **a**, **b** *USP33*, **c** miR-365, and **d** both *USP33* and miR-365a-3p simultaneously in SPC-A-1 and A549 cells. Each experiment was performed at least three times, and representative bands are shown

The above series of in vitro experiments suggested that miR-365a-3p promotes lung cancer progression by downregulating *USP33*; however, the signalling pathways involved in this process remain unknown. It has been reported that the USP33/SLIT2/ROBO1 signalling pathway inhibits the migration of cancer cells; therefore, we hypothesised that miR-365a-3p downregulates this pathway to promote lung cancer progression. To verify our hypothesis, a western blot assay was performed to observe changes in the expression of SLIT2 and ROBO1 in A549 and SPC-A-1 cells overexpressing *USP33* or miR-365a-3p alone or in combination. The results showed that the expression levels of SLIT2 and ROBO1 were upregulated following the overexpression of *USP33* (Fig. 5a, b) but downregulated following the overexpression of miR-365a-3p (Fig. 5c). However, when both miR-365a-3p and *USP33* were overexpressed, there was no significant change in the expression of SLIT2 or ROBO1 compared to levels in the control group (Fig. 5d). These results indicated that miR-365a-3p promotes lung cancer progression by downregulating the USP33/SLIT2/ROBO1 signalling pathway.

### miR-365a-3p promotes subcutaneous tumour formation in mice

**Fig. 6:**
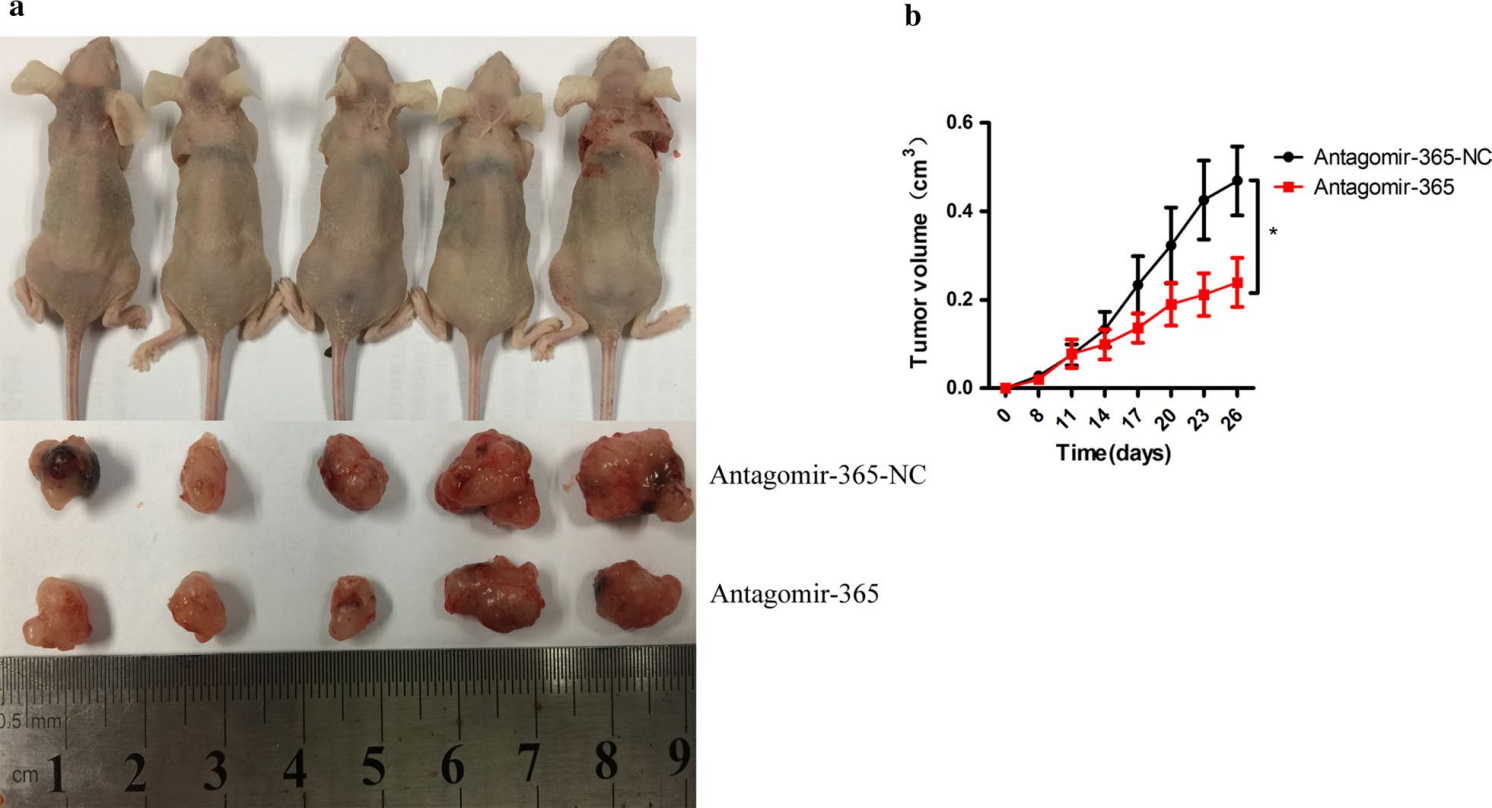
miR-365a-3p promotes subcutaneous tumour formation in mice. **a** Images show subcutaneous tumour growth at 26 days after the injection of A549 cells transfected with antagomiR-365 or control into the axilla of five BALB/c nude mouse xenograft models. Beginning 8 days after injection, tumour volumes (mm^3^) were measured every 3 days, and **b** time-dependent tumour growth curves were plotted. Data are expressed as mean ± SD

The results of this in vivo experiment showed that tumourigenic ability began to differ on day 8 post-injection. Beginning on day 8, tumour size changes were measured every 3 days using a slide rule, and tumour growth curves were plotted (Fig. 6). The tumour volumes on day 26 in mice injected with A549/antagomiR-NC cells were significantly larger than those in mice injected with A549/antagomiR-365. Our findings demonstrated that tumours derived from A549/antagomiR-365 cells grew more slowly than those derived from A549/antagomiR-NC cells. Thus, the results of the subcutaneous implantation tumour model further demonstrated that miR-365a-3p promotes tumour formation in vivo.

## Discussion

Identifying the mechanisms of lung cancer metastasis and methods for inhibiting metastasis is an important focus of the international medical community. MiRNAs play a dual role in tumour development and progression and participate in all aspects of tumour development [35–38]. Circulating miR-365a-3p may serve as a molecular marker for the diagnosis and prognostic evaluation of some tumours [39–41]. Thus, the role of miR-365a-3p in cancer should be further explored.

The regulation of miR-365a-3p differs among different tissues and genetic backgrounds, in which it can serve as either a tumour suppressor or oncogene [42, 43]. The dual nature of miR-365a-3p may be related to its unique topological structure, interactions with various signalling pathways, and induction of different biochemical reactions. It is therefore necessary to study the role of miR-365a-3p in specific tumour contexts. Although lung cancer is the most common cause of cancer-related deaths worldwide, the role of miR-365a-3p in the proliferation, invasion, and metastasis of lung cancer has not been previously reported. As lung adenocarcinoma is the main pathological type of lung cancer, it is important to study the role and regulation of miR-365a-3p in lung adenocarcinoma.

In this study, we found that miR-365a-3p was highly upregulated in cancer tissues and cell lines. Based on these experimental results, we speculated that miR-365a-3p may promote tumourigenesis in lung cancer. Functional assays demonstrated that miR-365a-3p promoted proliferation, migration, and invasion, further confirming our hypothesis. However, the pathway by which miR-365a-3p promotes lung cancer remained unknown. Thus, determining the role and mechanism of miR-365a-3p in lung adenocarcinoma was the first aim of this study.

Ubiquitination is a dynamic and reversible process, and the removal of ubiquitination modifications is mainly mediated by deubiquitinases, which are closely related to tumour occurrence and development [44–46]. In recent years, increasing attention has been paid to the role of deubiquitinases in tumour progression, and the molecular mechanisms underlying their regulation have gradually been clarified [47]. More than 40 deubiquitinating enzymes have been found to be associated with the occurrence and development of tumours. However, few studies have investigated the relationship between miRNAs and tumourigenesis in the context of deubiquitination. USP33 is a deubiquitinating enzyme that is closely related to the occurrence and development of tumours [28–30, 32]. The list of diseases currently reported to be associated with the dysregulation of USP33 includes breast cancer, acute lymphoblastic leukaemia, and lung cancer [28, 30, 48]. We sought to identify the downstream target genes of miR-365a-3p and found that *USP33* expression was strongly negatively correlated with that of miR-365a-3p in lung adenocarcinoma tissues. Functional rescue assays demonstrated that miR-365a-3p promoted proliferation, migration, and invasion by downregulating *USP33*. This is the first evidence for a lung cancer invasion and metastasis mechanism in which miR-365a-3p directly targets and downregulates *USP33*, which further promotes the proliferation, migration, and invasion activities of lung cancer. However, these results did not clarify the signalling pathway through which miR-365a-3p downregulates *USP33* to promote lung carcinogenesis.

Recently, an increasing number of studies have found that the SLIT2/ROBO1 signalling pathway is closely involved in tumourigenesis by inhibiting the proliferation, migration, and invasion of tumour cells [49–51]. USP33 deubiquitinates ROBO1 in lung cancer cells to maintain its stability, thereby regulating SLIT2 activity and inhibiting cancer cell metastasis [30]. Based on the above experiments, we hypothesised that miR-365a-3p may regulate the SLIT2/ROBO1 signalling pathway by targeting *USP33* to promote lung cancer. Based on western blot assay results, the expression levels of SLIT2 and ROBO1 were both downregulated following the overexpression of miR-365a-3p but restored when *USP33* was overexpressed at the same time. Combined with the above in vitro test results, we concluded that miR-365a-3p promotes lung cancer by downregulating the USP33/SLIT2/ROBO1 pathway.

To further validate this hypothesis, we performed in vivo experiments in a mouse lung cancer tumour model. A549/antagomiR-365 cells were injected into mice to observe subcutaneous tumour formation. The results suggested that the inhibition of miR-365a-3p expression reduced tumourigenicity. Thus, the in vivo experiment further demonstrated that miR-365a-3p plays a role in promoting tumour proliferation in lung adenocarcinoma.

## Conclusions

In summary, we not only confirmed that miR-365a-3p targets and downregulates *USP33* in lung adenocarcinoma, but we also further confirmed that miR-365a-3p promotes the proliferation, migration, and invasion of lung cancer cells by downregulating the USP33/SLIT2/ROBO1 pathway. To achieve early diagnosis and further improve the clinical outcomes of lung cancer, it is necessary to explore biomarkers for early detection and develop individualised treatment methods for lung cancer. Our elucidation of the mechanism by which miR-365a-3p mediates the USP33/SLIT2/ROBO1 signalling pathway in lung carcinogenesis thus provides a new strategy for the diagnosis and targeted therapy of lung cancer.

## Additional files

**Additional file 1.** Transfection efficiency of miR-365a-3p and *USP33*. (a) Transfection efficiency of agomiR-365 and antagomiR-365 in A549 and SPC-A-1 cells. (b) Transfection efficiency of pcDNA3.1-USP33 in A549 and SPC-A-1 cells.

**Additional file 2.** Association of USP33 expression with survival. (a) Survival analysis showing the association between high expression of USP33 and good prognosis.

**Additional file 3.** Construction of luciferase vector and identification of mutant *USP33* 3ʹ UTR by PCR. (a) *USP33* 3ʹ UTR PCR amplification product. (b) Positive clones identified by colony PCR. (c) psi-CHECK2 vector.

**Additional file 4: Table S1.** PCR sequences of miR-365a-3p and *USP33*. (a) hsa-miR-365a-3p sequence. (b) *USP33* 3ʹ UTR primer sequences.

## Abbreviations

miRNAs: microRNAs
NSCLC: non-small cell lung cancer
EMT: epithelial–mesenchymal transition
VDU1: VHL-interacting deubiquitinating enzyme 1
GAPDH: glyceraldehyde-3-phosphate dehydrogenase
SPF: specific pathogen-free
TCGA: The Cancer Genome Atlas
