# Supplementary figures and images for: MicroRNA-365 promotes lung carcinogenesis by downregulating the USP33/SLIT2/ROBO1 signalling pathway
Source: Cancer Cell Int. 2018 May 1;18:64. doi: 10.1186/s12935-018-0563-6 (PMC5930950; doi:10.1186/s12935-018-0563-6)

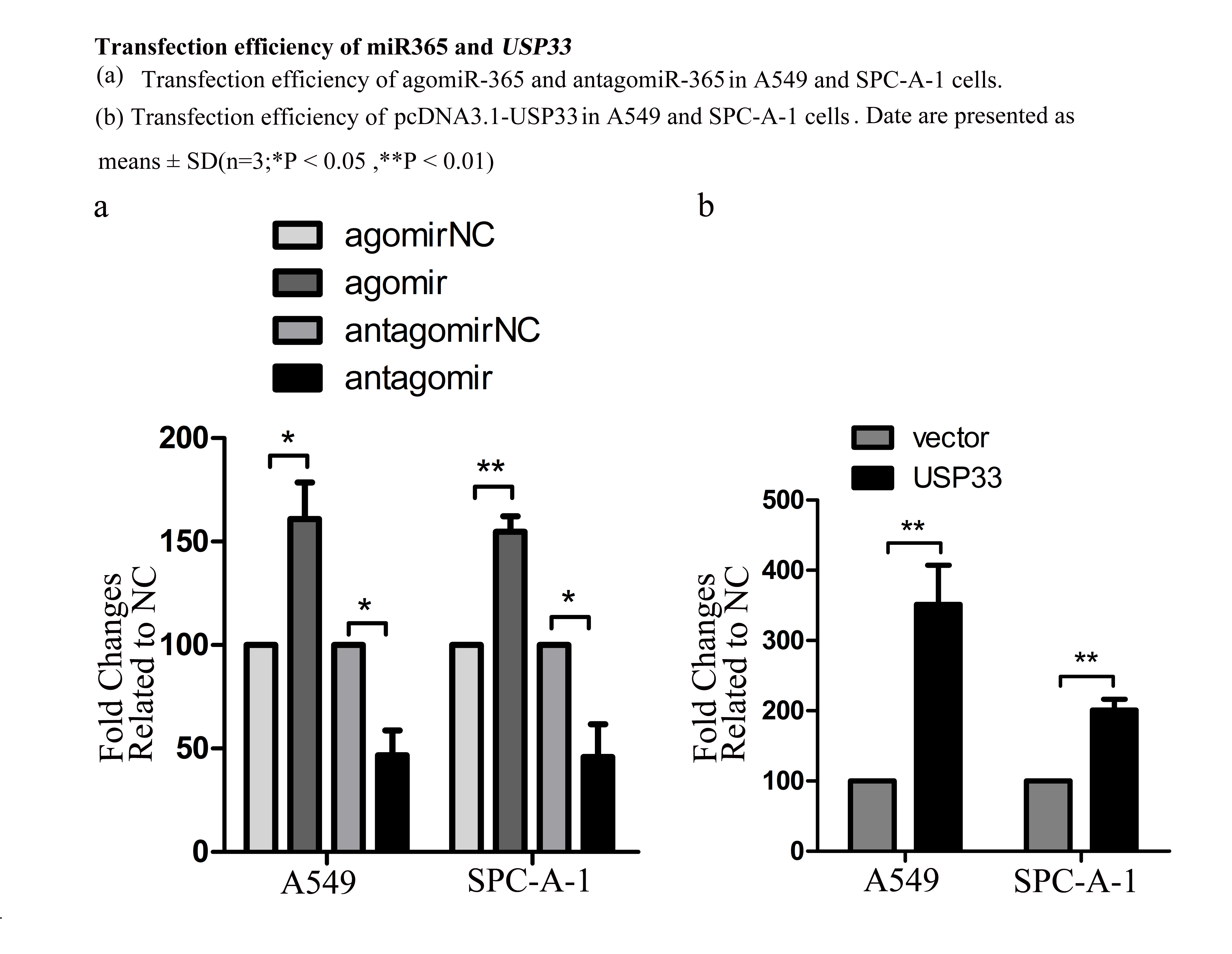

Supplement: Supplementary file 1 — Additional file 1. Transfection efficiency of miR-365a-3p and USP33. (a) Transfection efficiency of agomiR-365 and antagomiR-365 in A549 and SPC-A-1 cells. (b) Transfection efficiency of pcDNA3.1-USP33 in A549 and SPC-A-1 cells. [file 12935_2018_563_MOESM1_ESM.tif]

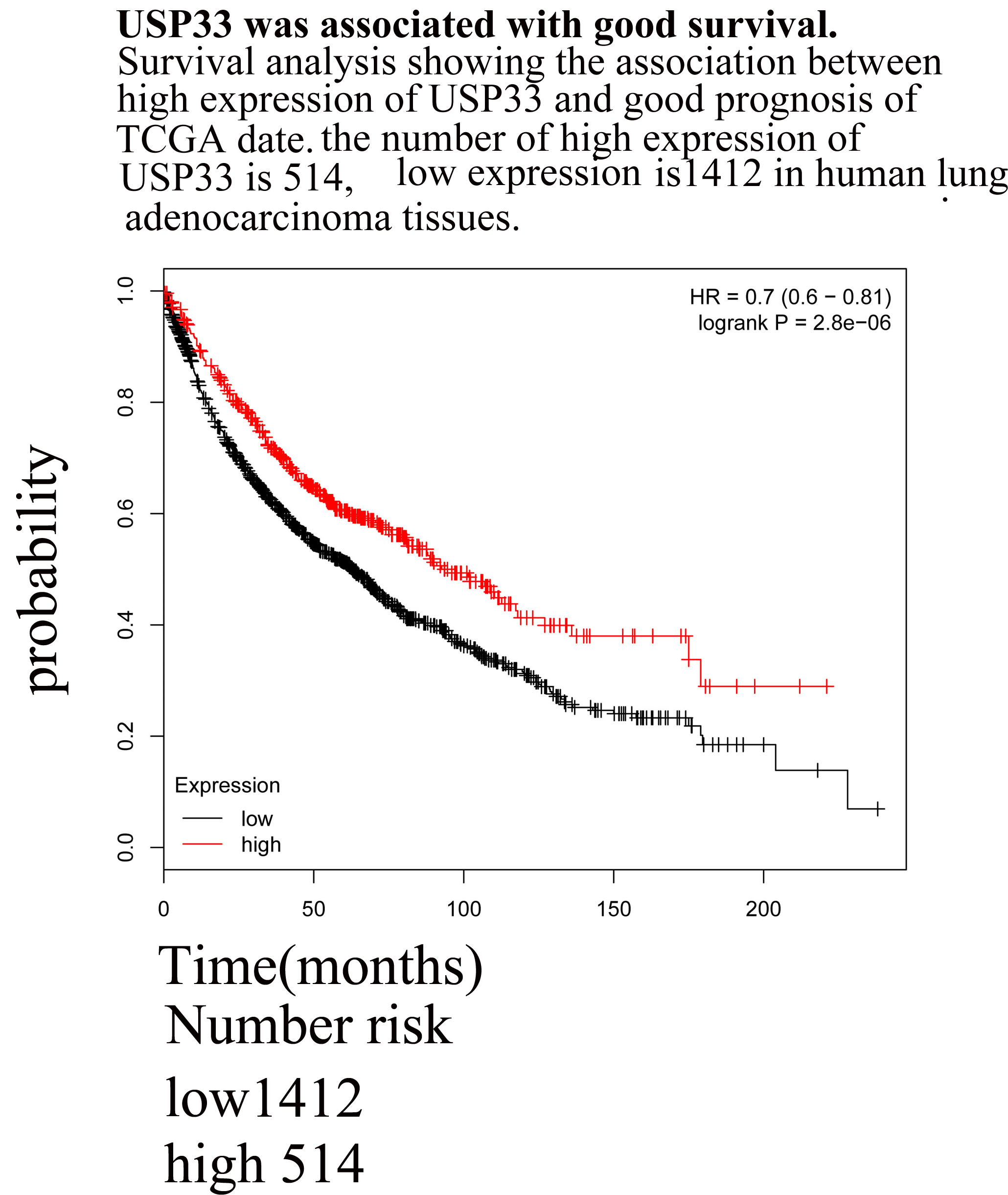

Supplement: Supplementary file 2 — Additional file 2. Association of USP33 expression with survival. (a) Survival analysis showing the association between high expression of USP33 and good prognosis. [file 12935_2018_563_MOESM2_ESM.tif]
