# Supplementary material for: MicroRNA-365 promotes lung carcinogenesis by downregulating the USP33/SLIT2/ROBO1 signalling pathway
Source: Cancer Cell Int. 2018 May 1;18:64. doi: 10.1186/s12935-018-0563-6 (PMC5930950; doi:10.1186/s12935-018-0563-6)
Supplement: Supplementary file 4 — Additional file 4: Table S1. PCR sequences of miR-365a-3p and USP33. (a) hsa-miR-365a-3p sequence. (b) USP33 3ʹ UTR primer sequences. [file 12935_2018_563_MOESM4_ESM.docx]

**A:**

**hsa-miR-365a-3p sequences：**

**UAAUGCCCCUAAAAAUCCUUAU**

**B:**

**Primer sequences**

**table1 PCR USP33-3’UTR primer** **sequences**

| Gene name | primer | **primer sequences** |
| --- | --- | --- |
| USP33-3’UTR | F | AAAAGTTTAAACTTTTTAGGATGTAGAGAGTTCTAATGAGGAATCAT |
|  | R | AAAAGCGGCCGCACATTTTAAAGACATTTTTATTGAGCTAATTTTAAC |
| USP33-3’UTR（Mut） | F | CTTCTTAGTCCCGTAAATGGAAGAATATATTAAAATGTGTAATATACCACAG |
|  | R | CTTCCATTTACGGGACTAAGAAGAAATAAATGGGATAAATGATG |
